# Eu-Doped Zeolitic Imidazolate Framework-8 Modified Mixed-Crystal TiO_2_ for Efficient Removal of Basic Fuchsin from Effluent

**DOI:** 10.3390/ma14237265

**Published:** 2021-11-27

**Authors:** Wanqi Zhang, Hui Liu, Zhechen Liu, Yuhong An, Yuan Zhong, Zichu Hu, Shujing Li, Zhangjing Chen, Sunguo Wang, Xianliang Sheng, Xiaotao Zhang, Ximing Wang

**Affiliations:** 1 College of Material Science and Art Design, Inner Mongolia Agricultural University, Hohhot 010018, China; nmgndcyyzwq@emails.imau.edu.cn (W.Z.); nndcyylh@emails.imau.edu.cn (H.L.); liuzhechen@emails.imau.edu.cn (Z.L.); anyuhong@emails.imau.edu.cn (Y.A.); zhongyuan@emails.imau.edu.cn (Y.Z.); lishujing@emails.imau.edu.cn (S.L.); 2 College of Science, Inner Mongolia Agricultural University, Hohhot 010018, China; hzc101@emails.imau.edu.cn (Z.H.); shengxl@iccas.ac.cn (X.S.); 3 Department of Sustainable Biomaterials, Virginia Polytechnic Institute and State University, Blacksburg, VA 24060, USA; chengo@vt.edu; 4 Sungro Bioresource & Bioenergy Technologies Corp., Edmonton, AB T6R3J6, Canada; wangsunguo@gmail.com; 5 Inner Mongolia Key Laboratory of Sandy Shrubs Fibrosis and Energy Development and Utilization, Hohhot 010018, China

**Keywords:** ZIF-8, Eu-doped, TiO_2_, photocatalysis, basic fuchsin

## Abstract

Zeolitic imidazolate framework-8 (ZIF-8) was doped with a rare-earth metal, Eu, using a solvent synthesis method evenly on the surface of a mixed-crystal TiO_2_(Mc-TiO_2_) structure in order to produce a core–shell structure composite ZIF-8(Eu)@Mc-TiO_2_ adsorption photocatalyst with good adsorption and photocatalytic properties. The characterisation of ZIF-8(Eu)@Mc-TiO_2_ was performed via X-ray diffraction (XRD), scanning electron microscopy (SEM), transmission electron microscopy (TEM), X-ray photoelectron spectroscopy (XPS), Brunauer–Emmett–Teller analysis (BET) and ultraviolet–visible light differential reflectance spectroscopy (UV-DRs). The results indicated that Eu-doped ZIF-8 was formed evenly on the Mc-TiO_2_ surface, a core–shell structure formed and the light-response range was enhanced greatly. The ZIF-8(Eu)@Mc-TiO_2_ for basic fuchsin was investigated to validate its photocatalytic performance. The effect of the Eu doping amount, basic fuchsin concentration and photocatalyst dosage on the photocatalytic efficiency were investigated. The results revealed that, when 5%-Eu-doped ZIF-8(Eu)@Mc-TiO_2_ (20 mg) was combined with 30 mg/L basic fuchsin (100 mL) under UV irradiation for 1 h, the photocatalytic efficiency could reach 99%. Further, it exhibited a good recycling performance. Thus, it shows certain advantages in its degradation rate and repeatability compared with previously reported materials. All of these factors suggested that, in an aqueous medium, ZIF-8(Eu)@Mc-TiO_2_ is an eco-friendly, sustainable and efficient material for the photocatalytic degradation of basic fuchsin.

## 1. Introduction

Dyes are widely used in various industries to colour industrial products, such as foods, cosmetics, leather, plastics and paper [1]. Large amounts of water containing these dyes are discharged in the environment, and these organic dyes present a threat to human and aquatic health because they are extremely water-soluble, nonbiodegradable, chemically stable and carcinogenic [2,3]. Basic fuchsin is a kind of triphenylmethane dye that is widely used in the dyeing of cotton, nylon, leather and paper [4]. Regular contact can affect the central nervous system, induce dizziness, drowsiness and muscle contractions, and can even cause cancer [5]. 

Several separation techniques, such as physical adsorption, electrochemical oxidation, biodegradation and Fenton oxidation, are used for the decolourisation and removal of these wastes from the water [6]. However, such traditional methods are often expensive and cause secondary pollution. Photocatalytic degradation is regarded as an ideal and economically feasible method because infinite solar energy can be applied to induce the redox reaction without any other type of energy resource [7]. To efficiently utilise solar energy to remove dyes from water, it is essential to design a highly active photocatalyst [8]. Thus far, many researchers have been extensively studying and testing various classes of photocatalytic materials in order to achieve the photochemical degradation of dyes [9].

Titanium dioxide (TiO_2_) is a nontoxic photocatalytic material with excellent physical and chemical properties, such as good stability and a strong oxidation ability [10]. The photocatalytic activity of TiO_2_ is mainly related to its crystal phase, morphology and size [11]. A large number of studies have confirmed that the photocatalytic activity of mixed-crystal TiO_2_ is better than that of single-crystal TiO_2_ [12]. Photogenerated electrons and holes can migrate from one phase to another through the two-phase interface, which can effectively separate the photogenerated electrons and holes, inhibit the recombination of the two and improve the quantum yield of the material [13]. Therefore, the preparation of mixed-crystal TiO_2_ micro/nanomaterials is an effective method for obtaining high-performance catalysts. In addition, the rapid recombination of photogenerated carriers is also an important factor hindering the enhancement of photocatalytic activity [14]. Therefore, avoiding the agglomeration of TiO_2_ and reducing the recombination of photogenerated carriers has become another research focus.

Metal–organic frameworks (MOFs) comprise a class of new porous materials that are widely used, especially in catalysis, adsorption, separation and gas storage, as well as in other fields [15]. A typical representative of zeolitic imidazolate frameworks (ZIFs), ZIF-8, has a metal imidazole unit bond length that is longer than that of the molecular sieve [16], and a pore cage diameter greater than that of the molecular sieve [17]. As a result, ZIF-8 has a larger specific surface area and pore size [18]. Many studies have reported that MOFs loaded on the surface of photocatalysts could not only increase the contact area between the photocatalyst and pollutants to adsorb organic dye and avoid the agglomeration of photocatalysts, but could also improve the degradation performance of photocatalysts [19,20]. 

Rare-earth (RE) metal doping or deposition is an effective method for photocatalytic performance [21]. RE metals are rich in 4f electronic conformation and can form complexes with Lewis bases, including organic acids, ethanol and thiols, thereby effectively changing the crystal structure, electronic conformation and optical properties [22,23]. RE ion doping not only resists the recombination of the photocatalyst’s photogenerated electrons and holes, but also broadens the spectral response range of photocatalysts and improves their photocatalytic ability [24]. Europium is the most active of RE metals. The electronic configuration of europium is [Xe] 4f^7^5s^2^5p^6^6s^2^. Due to the fact that the 4f orbital of Eu is incompletely filled, the electrons in this orbital are easily shielded by sub-outer electrons, resulting in the complex linear spectral properties of Eu ions. The introduction of Eu^3+^ ions into the matrix material can cause lattice defects of the matrix material, increase the surface-active sites of the material, improve the valence band position of the matrix material and increase the absorption of visible light. Thus, the resulting composite nanomaterials have a good performance in the fields of luminescence, new energy development and utilisation. In addition, there was no toxicity of europium compounds, and even some of the europium compounds could be used for future therapeutic alternative treatment strategies for some diseases [25]. Doping current photocatalytic materials with Eu is also considered a promising material preparation strategy for photocatalysts.

In this work, we prepare mixed-crystal structure TiO_2_(Mc-TiO_2_) and creatively distribute ZIF-8 doped by RE metal Eu using a solvent synthesis method evenly on the surface of the mixed-crystal TiO_2_ structure to produce a core–shell structure composite material ZIF-8(Eu)@Mc-TiO_2_ adsorption photocatalyst with good adsorption and photocatalytic properties. The photocatalytic performance of the synthesised ZIF-8(Eu)@Mc-TiO_2_ was investigated with basic fuchsin as the target degradation product of aerobic organic pollutants; satisfactory results were obtained. The effects of the Eu doping amount, basic fuchsin concentration and photocatalyst dosage on the photocatalytic efficiency of ZIF-8(Eu)@Mc-TiO_2_ for basic fuchsin were investigated, and its regeneration ability was examined through several photocatalytic cycles. Thus, ZIF-8(Eu)@Mc-TiO_2_ was found to exhibit a good recycling performance as well. The degradation rate of ZIF-8(Eu)@Mc-TiO_2_ for basic fuchsin could reach 99%, and, compared with previously reported materials, it shows certain advantages in terms of its degradation rate and repeatability. This study provides valuable theoretical explorations and technical experiments for the large-scale treatment of industrial wastewater containing basic fuchsin by ZIF-8(Eu)@Mc-TiO_2_.

## 2. Materials and Methods

### 2.1. Materials

Tetrabutyl titanate and 2-methylimidazole were purchased from Shanghai Macklin Biochemical Technology Co., Ltd. (Shanghai, China); N,N-dimethylformamide (DMF), anhydrous ethanol, polyethylene glycol 400 (PEG-400) and basic fuchsin were purchased from Tianjin Fuchen chemical reagent factory; europium nitrate hexahydrate was purchased from Tianjin Heowns Biochemical Technology Co., Ltd. (Tianjin, China); glacial acetic acid was purchased from Tianjin New Technology Industrial Park Kemao Chemical Reagent Co., Ltd. (Tianjin, China); and zinc nitrate hexahydrate was purchased from Chengdu Ai Keda Chemical Reagent Co., Ltd. (Chengdu, China)

### 2.2. Adsorption and Photocatalyst

#### 2.2.1. Preparation of Mixed-Crystal Structure TiO_2_(Mc-TiO_2_)

First, 10 mL absolute ethanol, 2.5 mL deionised water, 5 mL glacial acetic acid and 1 mL polyethylene glycol 400 (PEG-400) were placed into beaker A and 15 mL absolute ethanol was placed into beaker B. Then, 10 mL tetrabutyl titanate was slowly added to beaker A and stirred using a magnetic stirrer (HJ-6A, Changzhou, China) until the solution was well mixed. Then, the solution in beaker A was slowly added to beaker B and stirred vigorously to produce a transparent sticky gel. The sealed sol was placed in the beaker and placed in the shade for 24 h to form a soft elastic solid gel. The gel was removed from the sealing film and dried at 100 °C for 24 h in an electric drying oven (SY-101BS, Tianjin, China), and transparent granular crystals were obtained. After grinding, the powder was placed in a crucible and calcined at 700 °C in a muffle furnace (KSL-1100X, Hefei, China). After calcination, mixed-crystal structure TiO_2_(Mc-TiO_2_) was obtained through a standard sieve (Model Φ200).

#### 2.2.2. Preparation of Eu-Doped ZIF-8

As weighed by electronic balance (SQP, Beijing, China), 477.8 mg zinc nitrate hexahydrate, 120 mg 2-methylimidazole and a certain mass of europium nitrate hexahydrate were added into 36 mL N, N-dimethylformamide (DMF). The sample was fully dissolved and evenly distributed by an ultrasonic cleaner (KQ-300DE, Kunshan, China) for 20 min at room temperature. The mixed solution of zinc nitrate hexahydrate, 2-methylimidazole and europium nitrate hexahydrate was placed in the 100-mL polytetrafluoroethylene (PTFE) tank of a stainless-steel autoclave, heated to 140 °C at 5 °C/min in an electric drying oven (SY-101BS, Tianjin, China), kept for 24 h and then reduced to room temperature at a rate of 0.4 °C/min. The PTFE liner was removed and the yellow solid on the bottom and inner wall was scraped off and then washed and centrifuged with DMF. The final product was dried for 5 h at 60 °C in a vacuum drying oven (DZ-1BCⅡ, Tianjin, China) and ground into powder to obtain Eu-doped ZIF-8 (ZIF-8(Eu)).

#### 2.2.3. Preparation of ZIF-8(Eu)@Mc-TiO_2_

As weighed by electronic balance (SQP, Beijing, China), 477.8 mg zinc nitrate hexahydrate, 120 mg 2-methylimidazole and a certain mass of europium nitrate hexahydrate were added into 36 mL N, N-dimethylformamide (DMF), and 0.2 g of Mc-TiO_2_ was added. The sample was fully dissolved and evenly distributed by an ultrasonic cleaner (KQ-300DE, Kunshan, China) for 20 min at room temperature. The mixed solution of zinc nitrate hexahydrate, 2-methylimidazole, europium nitrate hexahydrate and Mc-TiO_2_ was placed in a 100 mL stainless-steel autoclave, heated to 140 °C at 5 °C/min in an electric drying oven (SY-101BS, Tianjin, China), kept for 24 h and then reduced to room temperature at a rate of 0.4 °C/min. The PTFE inner tank of the stainless-steel autoclave was removed. The yellow solid was scraped off the bottom and inner wall and then washed and centrifuged with DMF. The final product was dried at 60 °C in a vacuum drying oven (DZ-1BCⅡ, Tianjin, China) for 5 h and ground into powder. The amount of Eu doping in ZIF-8 is determined by the molar ratio of Eu to Zn.

#### 2.2.4. Evaluation of Photocatalytic Activity

At room temperature, a certain amount of ZIF-8(Eu)@Mc-TiO_2_ was added to 100 mL of a certain concentration of basic fuchsin solution. At first, the mixture was kept in darkness for 30 min in order to reach an adsorption–desorption balance between the photocatalyst and pollutant. The mixture was then transferred to a BL-GHX-V photocatalytic reactor and irradiated with a 550 W mercury lamp under air stirring. Subsequently, 5 mL of reaction solution was taken into the centrifuge tube every 20 min and centrifuged at 8000 rpm to remove the photocatalyst. The basic fuchsin solution concentration of the supernatant was determined by measuring the absorbance of the solution with a TU-1901 ultraviolet–visible spectrophotometer, and then the degradation rate was calculated by the absorbance at the maximum absorption wavelength. As shown in Figure S1 (in Supplementary Information), the maximum absorption wavelength is 543 nm. The degradation rate of the basic fuchsin was calculated as follows [26,27]:

*η* = 1 − *A_t_*/*A_0_* (1)

where *η* is degradation rate, and *A_0_* and *A_t_* are the absorbance before and after the solution was illuminated, respectively.

### 2.3. Characterization

The physicochemical properties of ZIF-8(Eu)@Mc-TiO_2_ were observed with different characterisation techniques. The specific surface area, pore structure and pore size of ZIF-8(Eu)@Mc-TiO_2_ were tested using V-Sorb 2800TP specific surface area and pore size analyser produced by Gold APP Instruments Corporation. Using an X-ray power diffractometer (XRD-700S) with Kα radiation in the 2θ range (5°–90°), X-ray diffraction (XRD) analyses of the ZIF-8, ZIF-8(Eu), TiO_2_ and ZIF-8(Eu)@Mc-TiO_2_ were performed, running at 40 kV and 100 mA. Scanning electron microscopy (SEM) of the ZIF-8(Eu)@Mc-TiO_2_ was performed using FEIQuanta650. The distribution of elements and the proportion of elements in the sample were analysed by energy-dispersive X-ray spectroscopy (EDX), and then the element composition of the sample was determined. Meanwhile, transmission electron microscopy (TEM) was performed using JEM-2100F (Tokyo, Japan) to determine the morphologies and structures of ZIF-8(Eu)@Mc-TiO_2_. TU-1950 dual-beam ultraviolet–visible spectrophotometry (UV-Vis) was performed to measure and depict the spectra of different powder samples (200–800 nm). The change in the light-response range before and after doping was analysed by fitting, and the spectral absorption threshold was determined. The AXIS-ULTRA DLD X-ray photoelectron spectrometer produced by Kratos company in UK was used for X-ray photoelectron spectroscopy (XPS) analysis. The binding energy of C1s (284.6 eV) was used to calibrate the binding energy.

## 3. Results

### 3.1. Crystal Structure Analysis of Photocatalyst

**Figure 1:**
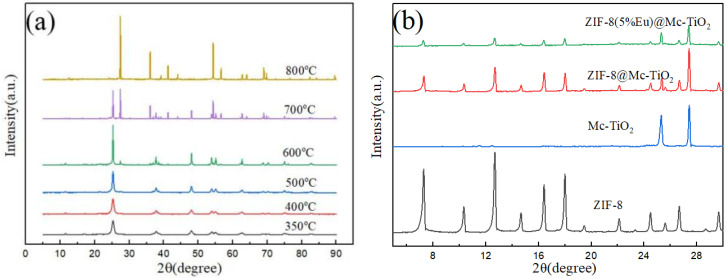
(**a**) XRD patterns of TiO_2_ at different temperatures and (**b**) XRD patterns of ZIF-8(Eu)@Mc-TiO_2_, ZIF-8@Mc-TiO_2_, Mc-TiO_2_, ZIF-8.

According to the diffraction angle corresponding to each diffraction peak in Figure 1a, compared with the standard diffraction cards (JCPDS No.21-1272 and JCPDS No.21-1276), it is found that different crystal forms and diffraction peak intensities appear after calcination at different temperatures. When the temperature range is 350–500 °C, the sample is anatase TiO_2_ with diffraction angles of (2θ) 25.21°, 37.82°, 47.9° and 62.56° belonging to the (101), (004), (200) and (204) crystal planes of anatase type, respectively [28]. As shown in Table S1, with the increase of temperature, the crystal size increases and the diffraction peak intensity of the main peak 25.21° also changes.It can be seen from Figure 1a that, when the temperature is 600–800 °C, the diffraction angles (2θ) are 27.4°, 36.0°, 41.56° and 54.26°, which correspond to the (110), (101), (111) and (211) crystal planes of rutile type, respectively, thus proving the presence of the rutile type [29]. The mixed-crystal structure appears at 600 °C, and especially at 700 °C, which belongs to anatase and rutile crystal. When the temperature reaches 800 °C, the main peak of anatase disappears, but the small peak of anatase still remains. The mixed-crystal structure is particularly obvious at 700 °C, but, at 600 °C and 800 °C, the mixed-crystal structure is not obvious and the hybrid degree of crystal forms is low. Therefore, the temperature used to calculate the proportion of different crystal forms of the mixed-crystal structure is 700 °C. The rutile type and anatase type occupy 48.38% and 51.62% of the crystal, respectively, at 700 °C. 

As shown by the black line in Figure 1b, the strong diffraction peaks at 7.35°, 10.34°, 12.68°, 14.66°, 16.5° and 18.0° correspond to the (011), (002), (112), (022), (013) and (222) crystal planes of ZIF-8, respectively. The blue line in Figure 1b indicates the diffraction peaks at 25.21° and 27.4°, corresponding to the (101) crystal plane of anatase TiO_2_ and the (110) rutile crystal plane of rutile TiO_2_ in the Mc-TiO_2_, respectively. The peak of composite ZIF-8@Mc-TiO_2_ and ZIF-8(Eu)@Mc-TiO_2_ is the composite peak of Mc-TiO_2_ and ZIF-8(Eu), which proves that the material is a composite of both. Due to the different intensities of the peaks, individual peaks are masked [30]. And the XRD patterns of ZIF-8 (Eu) @Mc-TiO_2_ doped with different contents of Eu was in Supplementary Material Figure S2 (in Supplementary Information).

### 3.2. Morphology Analysis

**Figure 2:**
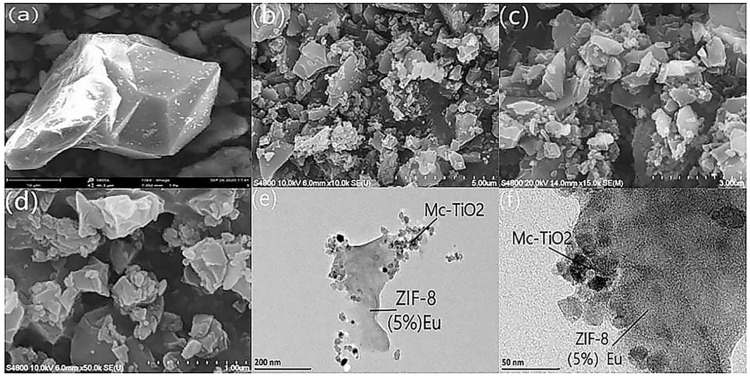
SEM (**a**) of ZIF-8, SEM (**b**–**d**) and TEM (**e**,**f**) of ZIF-8 (5%Eu)@Mc-TiO_2_.

The morphology of ZIF-8 and ZIF-8(5%Eu)@Mc-TiO_2_ was observed by scanning electron microscopy (SEM) and transmission electron microscopy (TEM). Figure 2a shows that bare ZIF-8 with a smooth surface and a sharp angular appearance presents a rhombic dodecahedron shape. Figure 2b–d shows that ZIF-8(5%Eu)@Mc-TiO_2_ is distributed in layers and blocks. As shown in Figure 2e–f, ZIF-8(5%Eu) is uniformly grown on the surface of Mc-TiO_2_. The formation of the ZIF-8(5%Eu)@Mc-TiO_2_ composite attenuates the agglomeration of TiO_2_ and also improves the catalytic ability.

### 3.3. Element Analysis

**Figure 3:**
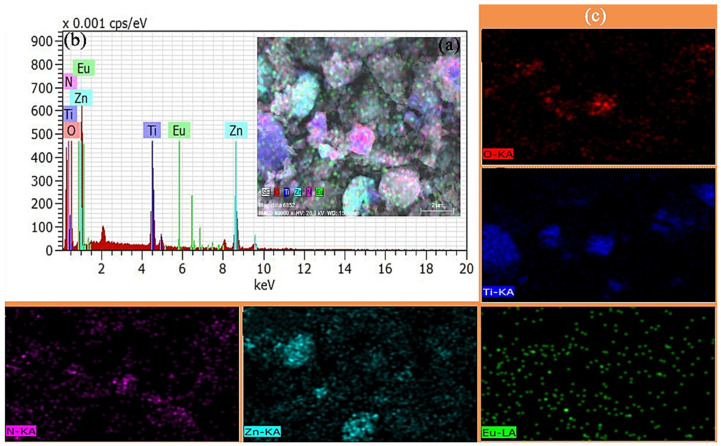
(**a**,**b**) ZIF-8(Eu)@Mc-TiO_2_ EDX diagram and (**c**) single element EDX mapping of ZIF-8(Eu)@Mc-TiO_2_.

The elemental composition of the ZIF-8(Eu)@Mc-TiO_2_ micron composite was detected by energy-dispersive X-ray spectroscopy (EDX) mapping. As shown in Figure 3a, the ZIF-8 are uniformly grown on the surface of TiO2, which indicates that the ZIF-8 successfully coated TiO_2_. The formation of the ZIF-8(Eu)@Mc-TiO_2_ micron composite greatly attenuates the agglomeration of TiO_2_ and also improves the catalytic ability. As shown in Figure 3a, there are five elements that are dispersed on the surface of the material that could also be seen from Figure 3b, which are: oxygen, titanium, zinc, europium and nitrogen on the surface of the ZIF-8(Eu)@Mc-TiO_2_. The red, blue, green, cyan, and purple colours are the elemental distributions of O, Ti, Eu, Zn and N, respectively. Figure 3c shows the mapping of every single element. As shown in the O and Ti mapping, the distributions of O and Ti are similar. This demonstrates that Ti–O is effectively combined into TiO_2_, which is verified by XRD. The Eu and Zn mapping also shows the distribution of Eu, which is the same as that of Zn, indicating that Eu is doped into ZIF-8. A comparison of the mapping of Ti and Zn shows that their distributions in the composite are different and overlap. This is because the distribution of Mc-TiO_2_ in the composite is uneven. Some Mc-TiO_2_ agglomerates and ZIF-8(Eu) do not completely cover Mc-TiO_2_.

**Figure 4:**
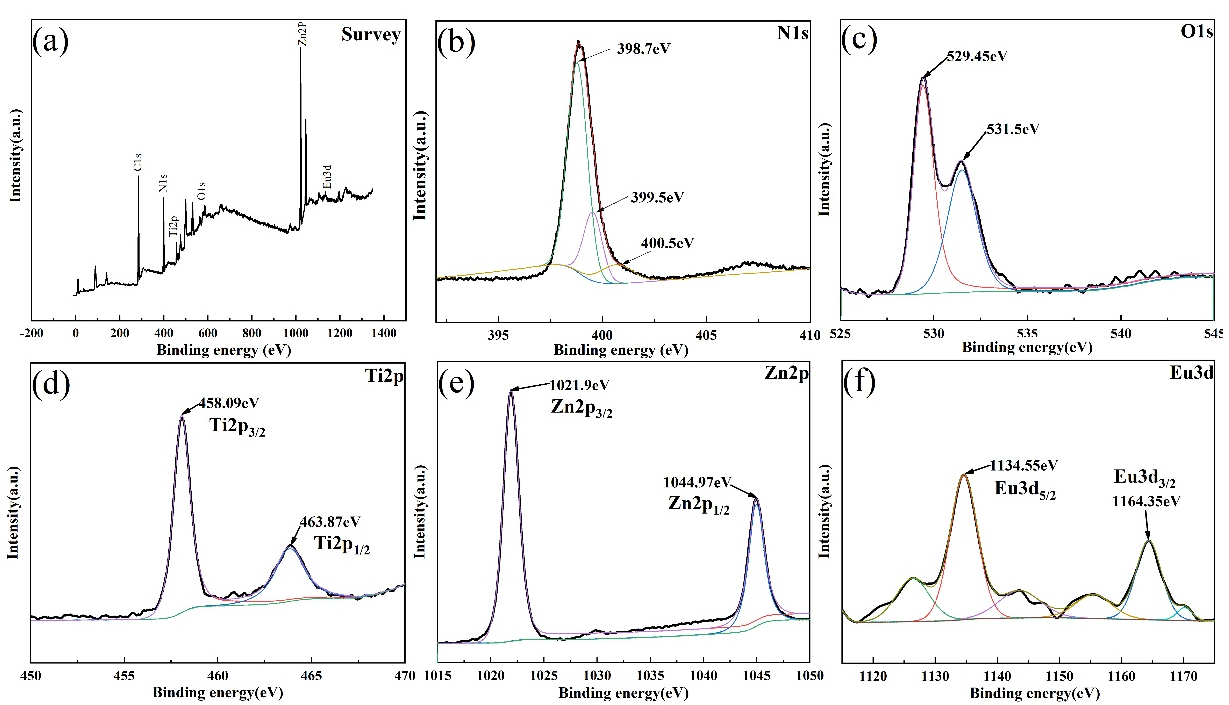
(**a**) XPS survey spectrum of ZIF-8(Eu)@Mc-TiO_2_. (**b**–**f**) N 1s, O 1s, Ti 2p, Zn 2p, Eu 3d, XPS spectrum of ZIF-8(Eu)@Mc-TiO_2_.

Figure 4 shows the spectra using the X-ray photoelectron spectroscopy (XPS) technique to analyse the valence state of various elements in hybrid. It can be seen from Figure 4a that nitrogen, titanium, oxygen, zinc, europium and other elements are detected in the composite. Figure 4b is the XPS diagram of N in the composite, which belongs to ZIF-8, and there are three peaks at 398.7 eV, 399.5 eV and 400.5 eV corresponding to the pyridine-type, pyrrole-type and graphite-type nitrogen of the N1s peak. Pyridine-type nitrogen atoms replace the carbon atoms in the six-member ring, and pyrrole-type nitrogen atoms are located in the five-member ring [31]. Pyridine-type and pyrrole-type nitrogen can enhance the adsorption performance of ZIF-8 [32]. The XPS spectrum of Ti 2p from the ZIF-8(Eu)@Mc-TiO_2_ composite displays two peaks at 458.09 eV and 464.87 eV, which correspond to the binding energies of Ti 2p_1/2_ and Ti 2p_3/2_, respectively, in Figure 4d. This indicates the presence of the oxidation state Ti4þ in the ZIF-8(Eu)@TiO_2_ composite. Compared to the standard TiO_2_ sample, the Ti 2p_1/2_ and Ti 2p_3/2_ binding energy values of the ZIF-8(Eu)@TiO_2_ composite become larger, indicating that the electrons in the TiO_2_ are transferred to ZIF-8; this may be due to the fact that the doping of Eu causes a change in the chemical environment around the Ti atom of the electron cloud, finally changing the electron binding energy. This novel structure might induce charge transfer, thereby promoting photoelectron hole separation. Figure 4c shows the XPS of oxygen in the composite. Due to the fact that the molecular formula of ZIF-8 is C_8_H_10_N_4_Zn, the oxygen is considered to have come from the oxygen atoms in TiO_2_. After peak fitting, it is found that the diffraction peaks appear at 529.45 eV and 531.5 eV. These correspond to lattice oxygen and surface adsorbed oxygen, respectively. As can be seen from Figure 4e, the Zn 2p_3/2_ and Zn 2p_1/2_ binding energies of the composite are 1021.9 eV and 1044.97 eV, respectively [33]. It is proven that Zn exists in the composite in the form of a +2 valence. Figure 4f is the XPS peak fitting diagram of Eu, where 1134.55 eV and 1164.35 correspond to Eu 3d_5/2_ and Eu 3d_3/2_, indicating that doped Eu mainly exists in the form of a +3 valence. In ZIF-8(Eu)@Mc-TiO_2_, the valence of each element did not change.

### 3.4. Analysis of Specific Surface Area

**Figure 5:**
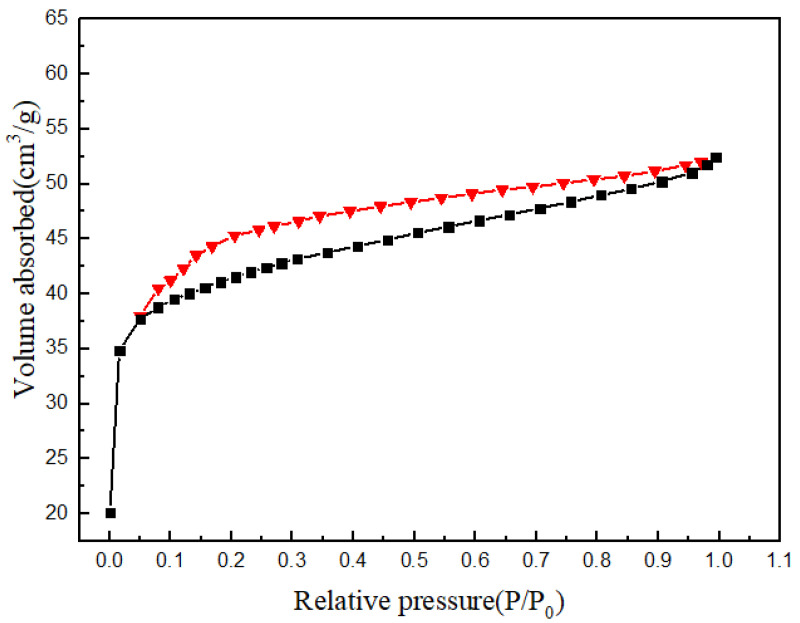
N_2_ desorption diagram of ZIF-8(Eu)@Mc-TiO_2_.

Figure 5 is the N_2_ adsorption–desorption curve of ZIF-8(Eu)@Mc-TiO_2_. It can be seen from Figure 5 that the N_2_ adsorption–desorption curve of ZIF-8(Eu)@Mc-TiO_2_ is a type-IV IUPAC curve, and that there is single-layer adsorption of capillary condensation. When *P*/*P_0_* is between 0.1 and 1.0, there is a retention loop and capillary condensation [34]. The retention loop is H4-type. It can be seen from Table 1 that the Brunauer–Emmett–Teller (BET) specific surface area, total pore volume (*P*/*P_0_* = 0.99) and average pore diameter of ZIF-8(Eu)@Mc-TiO_2_ are 159.23 m^2^/g, 0.0808 cm^3^/g and 2.0305 nm, respectively; the average pore diameter is in the mesoporous range.

### 3.5. Analysis of UV-Vis Diffuse Reflectance Spectroscopy (UV-Vis DRS)

**Figure 6:**
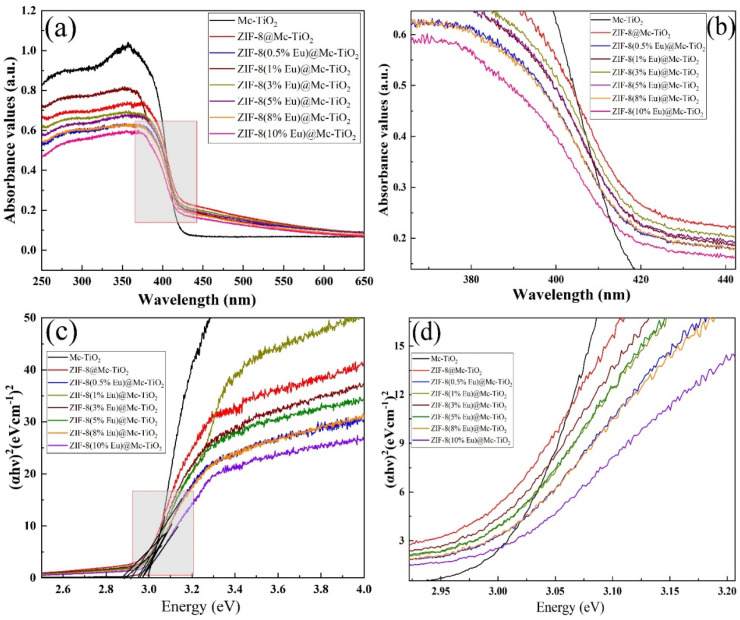
UV-Vis DRS diagram (**a**,**b**) and band gap width diagram (**c**,**d**).

The band gaps of anatase and rutile TiO_2_ are 3.2 eV and 3.0 eV, respectively. When irradiated by ultraviolet light, photogenerated electrons will transition from the valence band to the conduction band. At this time, the light energy is converted into electron energy, so that the electron can cross the forbidden band and the photogenerated electron can be transferred to form the photogenerated electron–hole pair [35]. Figure 6a,b shows that ZIF-8 is doped with different amounts of the Eu composite Mc-TiO_2_, where (b) is the enlarged shadow of (a). Figure 6c,d show the band gap of the above materials, where (d) shows the enlarged shadow of (c). Table 2 lists the band gap values of the materials. It can be seen from Figure 6a that, with the doping of EuNO_3_·6H_2_O, the photoresponse range increases and the UV diffuse reflectance spectrum curve is red-shifted, which causes the photoresponse range to increase. As can be seen from Table 2, the band gaps of ZIF-8@Mc-TiO_2_ and ZIF-8(Eu)@Mc-TiO_2_ have been reduced by different degrees because ZIF-8 contains light-absorbing organic chromophores and photo-excited semiconductor metal clusters [36]. The charge-transfer interaction between organic groups as donors and Zn^4+^ ions as acceptors is longer than that of organic and inorganic groups. Organic molecules can be used as a photon antenna to inject metal quantum dots through energy transfer for photosensitisation in order to improve the light-response range [37].

### 3.6. Evaluation of Photocatalyst Performance

#### 3.6.1. Effect of Eu Doping Amount for Photocatalytic Efficiency

**Figure 7:**
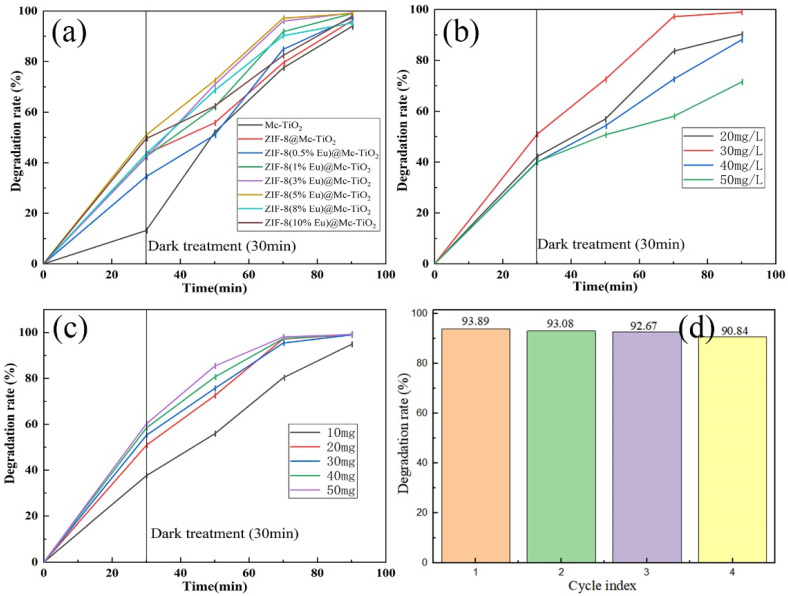
(**a**) ZIF-8(Eu)@Mc-TiO_2_ and Mc-TiO_2_ photo catalytic efficiency diagram, (**b**) effect of initial concentration of basic fuchsin on catalytic efficiency, (**c**) effect of catalyst dosage on catalytic efficiency and (**d**) recycling times of catalyst.

A suitable Eu doping amount plays an important role in exerting the catalytic ability of the ZIF-8(Eu)@Mc-TiO_2_ photocatalyst. Therefore, we conducted an experiment in which Eu was doped from 0% to 10% in order to determine the appropriate amount of Eu. It can be seen from Figure 7a that the catalytic efficiency is the highest when the Eu doping amount is 5%. The degradation efficiency can reach 99% under UV irradiation for 1 h. From the overall trend of photocatalytic efficiency, the photocatalytic efficiency of 5% Eu-doped materials is better than that of other materials. It is possible that the TiO_2_ sample at 700 °C has two crystal forms. The combination of the two crystal forms is equivalent to the combination of two semiconductors, which causes the transfer of photogenerated electrons and holes, inhibits the combination of photogenerated electrons and holes and produces more free radicals, thus improving the photocatalytic efficiency. In addition, Eu doping increases the active sites on the surface of the material. When Eu doping is excessive, the pore structure of ZIF-8 will be blocked, the specific surface area of ZIF-8 will be decreased, the adsorption performance will be reduced and the photocatalytic performance of ZIF-8 will be reduced [38].

The catalytic conditions of ZIF-8(Eu)@Mc-TiO_2_ are the same as those of Mc-TiO_2_, whereas the catalytic efficiency of ZIF-8(Eu)@Mc-TiO_2_ is higher than that of Mc-TiO_2_. ZIF-8 with 5% Eu doping has an especially greater catalytic efficiency, which is mainly due to the increase in the specific surface area and the introduction of more reaction sites. The increase in the specific surface area solves the problem of insufficient reaction sites in Mc-TiO_2_. ZIF-8 provides a large number of reaction sites for the photocatalytic reaction and improves the photocatalytic efficiency [39]. At the same time, with the addition of Eu, the photoresponse range increases by varying degrees, which increases the utilisation of light and improves the photocatalytic efficiency [40].

#### 3.6.2. Effect of Basic Fuchsin Concentration for Photocatalytic Efficiency

Figure 7b shows the effect of the basic fuchsin solution concentration on the catalytic efficiency. It can be seen from Figure 7b that, with an increase in the solution concentration from 20 mg/L to 50 mg/L, the catalytic efficiency first increases and then decreases, and the catalytic efficiency of the basic fuchsin solution at 30 mg/L is the highest. Due to the presence of photogenerated carriers on the surface of the composite, the 20 mg/L basic fuchsin solution cannot make full use of the reaction sites [41]. When the concentration reaches 30 mg/L, the reaction sites are fully utilised and the photocatalytic efficiency is improved. When the concentration of the solution increases, the light-penetration ability decreases, resulting in decreases in the light response, photogenerated carriers and photocatalytic efficiency.

#### 3.6.3. Effect of Dosage of ZIF-8(Eu)@Mc-TiO_2_ for Photocatalytic Efficiency

Figure 7c shows the effect of the catalyst dosage on the catalytic efficiency, where it can be seen that the photocatalytic efficiency increases when increasing the catalyst dosage from 10 mg to 50 mg, and that there is a positive correlation between the catalyst dosage and photocatalytic efficiency. It is possible that the specific surface area and reaction sites of the materials are improved with the increase in the amount of the catalyst. Under certain other conditions, as the amount of catalyst increases, the amount of photogenerated electron holes is increased, and enough superoxide radicals and hydroxyl radicals are produced to participate in the degradation reaction [42].

#### 3.6.4. Reuse

In order to investigate the stability and reusability of ZIF-8(Eu)@Mc-TiO_2_, four cycles of photodegradation were performed under the same conditions. After each photodegradation experiment, ZIF-8(Eu)@Mc-TiO_2_ was collected, washed with anhydrous alcohol and deionised water and dried in a vacuum oven at 100 °C; then, the same sample was subjected to the next photodegradation experiment. Figure 7d shows the stability and reusability results of the ZIF-8(Eu)@Mc-TiO_2_ composite. The degradation rate of ZIF-8(Eu)@Mc-TiO_2_ decreases slightly because of the unavoidable loss during the recovery process. In the four cycles of the photodegradation experiment, the catalysts maintained excellent degradation activity. The removal rate of basic fuchsin dropped from 93.89% in the first test to 90.84% in the fifth test. This result demonstrates that ZIF-8(Eu)@Mc-TiO_2_ has excellent stability and reusability, and that the internal interactions of the composite allow it to maintain its original high efficiency. As shown in Table 3, the degradation rate of the ZIF-8(Eu)@Mc-TiO_2_ composite for basic fuchsin was higher than that of other materials.

## 4. Conclusions

In this paper, TiO_2_(Mc-TiO_2_) with a mixed-crystal structure of anatase and rutile types was prepared by a sol-gel method at a calcination temperature of 700 °C. ZIF-8 doped with Eu was compounded with Mc-TiO_2_ using the solvothermal method to obtain ZIF-8(Eu)@Mc-TiO_2_. XRD, SEM, TEM, XPS, UV-Vis DRS, BET specific surface area analysis and photocatalytic experiments were carried out. The results show that the specific surface area and the light-response range of the Mc-TiO_2_ composite ZIF-8 are increased. After UV irradiation for 1 h, 20 mg of ZIF-8(Eu)@Mc-TiO_2_ catalysed 100 mL of 30 mg/L basic fuchsin solution at a degradation rate of up to 99%, demonstrating an excellent photocatalytic effect. In addition, the catalytic efficiency was 90.84% after four cycles. All of the results demonstrate that ZIF-8(Eu)@Mc-TiO_2_ is an efficient photocatalytic material for the degradation of organic dyes.

## Figures and Tables

**Table 1:** ZIF-8(Eu)@Mc-TiO_2_-specific surface area and pore size values.

Sample	SBET (m^2^/g)	V_Pore_ (cm^3^/g)	R_Ave_ (nm)
ZIF-8(Eu)@Mc-TiO_2_	159.23	0.0808	20,305

**Table 2:** List of material forbidden band widths.

Sample	Mc-TiO_2_	ZIF-8@Mc-TiO_2_	ZIF-8(0.5%Eu)@Mc-TiO_2_	ZIF-8(1%Eu)@Mc-TiO_2_	ZIF-8(3%Eu)@Mc-TiO_2_	ZIF-8(5%Eu)@Mc-TiO_2_	ZIF-8(8%Eu)@Mc-TiO_2_	ZIF-8(10%Eu)@Mc-TiO_2_
Eg(eV)	2.97	2.94	2.92	2.94	2.92	2.90	2.85	2.83

**Table 3:** ZIF-8(Eu)@Mc-TiO_2_-specific surface area and pore size values.

Materials	Basic Fuchsin Concentration	Degradation Rate
Fe_2_O_3_/g-C_3_N_4_ [43]	25 mg/L	92%
Ag/AgVO_3_ [44]	20 mg/L	93.6%
H_4_SiW_6_Mo_6_O_4O_/SiO_2_ [45]	8 mg/L	98%
Fe_3_O_4_/SiO_2_ core-shell nanoparticles [46]	10 mg/L	98%
This work	30 mg/L	99%

## Data Availability

Date sharing not applicable.

## Supplementary Materials

The following are available online at https://www.mdpi.com/article/10.3390/ma14237265/s1, Table S1: TiO_2_ grain sizes at different temperatures, Figure S1: Wavelength scanning curve of basic fuchsin, Figure S2: XRD patterns of ZIF-8 (Eu) @Mc-TiO_2_ doped with different contents of Eu.

## References

[1] Rai H.S., Bhattacharyya M.S., Singh J., Bansal T., Vats P., Banerjee U. (2005). Removal of dyes from the effluent of textile and dyestuff manufacturing industry: A review of emerging techniques with reference to biological treatment. Crit. Rev. Environ. Sci. Technol..

[2] Shindy H. (2017). Fundamentals in the chemistry of cyanine dyes: A review. Dye. Pigment..

[3] Yamjala K., Nainar M.S., Ramisetti N.R. (2016). Methods for the analysis of azo dyes employed in food industry—A review. Food Chem..

[4] Mali P., Mahajan M., Patil D., Kulkarni M. (2000). Biodecolourisation of members of triphenylmethane and azo group of dyes. J. Sci. Ind. Res..

[5] Jain R., Mendiratta S., Kumar L., Srivastava A. (2021). Green synthesis of iron nanoparticles using Artocarpus heterophyllus peel extract and their application as a heterogeneous Fenton-like catalyst for the degradation of Fuchsin Basic dye. Curr. Res. Green Sustain. Chem..

[6] Rashid R., Shafiq I., Akhter P., Iqbal M.J., Hussain M. (2021). A state-of-the-art review on wastewater treatment techniques: The effectiveness of adsorption method. Environ. Sci. Pollut. Res..

[7] Khan K., Tareen A.K., Aslam M., Sagar R.U.R., Zhang B., Huang W., Mahmood A., Mahmood N., Khan K., Zhang H. (2020). Recent progress, challenges, and prospects in two-dimensional photo-catalyst materials and environmental remediation. Nano-Micro Lett..

[8] Busuioc-Tomoiagă A., Mertens M., Cool P., Bilba N., Vansant E. (2008). A simple way to design highly active titania/mesoporous silica photocatalysts. Studies in Surface Science and Catalysis.

[9] Akpan U.G., Hameed B.H. (2009). Parameters affecting the photocatalytic degradation of dyes using TiO2-based photocatalysts: A review. J. Hazard. Mater..

[10] Yemmireddy V.K., Hung Y.-C. (2015). Effect of binder on the physical stability and bactericidal property of titanium dioxide (TiO_2_) nanocoatings on food contact surfaces. Food Control.

[11] Serga V., Burve R., Krumina A., Romanova M., Kotomin E.A., Popov A.I. (2021). Extraction–Pyrolytic Method for TiO2 Polymorphs Production. Crystals.

[12] Zhao Z., Zhao X., Zhang M., Sun X. (2021). Charge-Transfer Process in Surface-Enhanced Raman Scattering Based on Energy Level Locations of Rare-Earth Nd^3+^-Doped TiO_2_ Nanoparticles. Nanomaterials.

[13] Tsebriienko T., Popov A.I. (2021). Effect of poly (titanium oxide) on the viscoelastic and thermophysical properties of interpenetrating polymer networks. Crystals.

[14] Qian R., Zong H., Schneider J., Zhou G., Zhao T., Li Y., Yang J., Bahnemann D.W., Pan J.H. (2019). Charge carrier trapping, recombination and transfer during TiO_2_ photocatalysis: An overview. Catal. Today.

[15] Xu X., Zhang Z., Wang X. (2015). Well-defined metal–organic-framework hollow nanostructures for catalytic reactions involving gases. Adv. Mater..

[16] Lee Y.-R., Jang M.-S., Cho H.-Y., Kwon H.-J., Kim S., Ahn W.-S. (2015). ZIF-8: A comparison of synthesis methods. Chem. Eng. J..

[17] Pan Y., Liu Y., Zeng G., Zhao L., Lai Z. (2011). Rapid synthesis of zeolitic imidazolate framework-8 (ZIF-8) nanocrystals in an aqueous system. Chem. Commun..

[18] Jiang X., He S., Han G., Long J., Li S., Lau C.H., Zhang S., Shao L. (2021). Aqueous one-step modulation for synthesizing monodispersed ZIF-8 nanocrystals for mixed-matrix membrane. ACS Appl. Mater. Interfaces.

[19] Li X., He W., Li C., Song B., Liu S. (2021). Synergetic surface modulation of ZnO/Pt@ ZIF-8 hybrid nanorods for enhanced photocatalytic CO_2_ valorization. Appl. Catal. B Environ..

[20] Zhao K., Zhang Z., Feng Y., Lin S., Li H., Gao X. (2020). Surface oxygen vacancy modified Bi_2_MoO_6_/MIL-88B (Fe) heterostructure with enhanced spatial charge separation at the bulk & interface. Appl. Catal. B Environ..

[21] Serga V., Burve R., Krumina A., Pankratova V., Popov A.I., Pankratov V. (2021). Study of phase composition, photocatalytic activity, and photoluminescence of TiO_2_ with Eu additive produced by the extraction-pyrolytic method. J. Mater. Res. Technol..

[22] Reszczyńska J., Grzyb T., Sobczak J.W., Lisowski W., Gazda M., Ohtani B., Zaleska A. (2014). Lanthanide co-doped TiO_2_: The effect of metal type and amount on surface properties and photocatalytic activity. Appl. Surf. Sci..

[23] Sivasankari J., Sankar S., Selvakumar S., Vimaladevi L., Krithiga R. (2014). Synthesis, structural and optical properties of Er doped, Li doped and Er+ Li co-doped ZnO nanocrystallites by solution-combustion method. Mater. Chem. Phys..

[24] Dhiman M., Chudasama B., Kumar V., Tikoo K., Singhal S. (2019). Augmenting the photocatalytic performance of cobalt ferrite via change in structural and optical properties with the introduction of different rare earth metal ions. Ceram. Int..

[25] Patra C.R., Moneim S.S.A., Wang E., Dutta S., Patra S., Eshed M., Mukherjee P., Gedanken A., Shah V.H., Mukhopadhyay D. (2009). In vivo toxicity studies of europium hydroxide nanorods in mice. Toxicol. Appl. Pharmacol..

[26] Ma R., Zhang S., Wen T., Gu P., Li L., Zhao G., Niu F., Huang Q., Tang Z., Wang X. (2019). A critical review on visible-light-response CeO_2_-based photocatalysts with enhanced photooxidation of organic pollutants. Catal. Today.

[27] Wei Q., Wang S., Li W., Yuan X., Bai Y. (2015). Hydrophobic ZnO-TiO_2_ nanocomposite with photocatalytic promoting self-cleaning surface. Int. J. Photoenergy.

[28] Zhu H.Q., Lou C.Y., Liu F.R., Jiang G.J., Ye X.Y., Yuan R.H. (2021). Nanosized S-Doped TiO_2_ with Effective Visible-Light Degradation of BTEX from Wood-Based Panels. Key Engineering Materials.

[29] Hou J., Wang Y., Zhou J., Lu Y., Liu Y., Lv X. (2021). Photocatalytic degradation of methylene blue using a ZnO/TiO_2_ heterojunction nanomesh electrode. Surf. Interfaces.

[30] Chen J., Guo Y., Kang T., Liu X., Wang X., Zhang X. (2021). In Situ Growth of ZIF-8 Nanocrystals on the Pore Walls of 3D Ordered Macroporous TiO_2_ for a One-Pot Cascade Reaction. Catalysts.

[31] Tuncel D., Ökte A. (2021). Improved adsorption capacity and photoactivity of ZnO-ZIF-8 nanocomposites. Catal. Today.

[32] Parkash A. (2020). Copper doped zeolitic imidazole frameworks (ZIF-8): A new generation of single-atom catalyst for oxygen reduction reaction in alkaline media. J. Electrochem. Soc..

[33] Hu C., Huang Y.-C., Chang A.-L., Nomura M. (2019). Amine functionalized ZIF-8 as a visible-light-driven photocatalyst for Cr (VI) reduction. J. Colloid Interface Sci..

[34] Zhang W., An Y., Li S., Liu Z., Chen Z., Ren Y., Wang S., Zhang X., Wang X. (2020). Enhanced heavy metal removal from an aqueous environment using an eco-friendly and sustainable adsorbent. Sci. Rep..

[35] Wang S., Tang S., Gao H., Chen X., Liu H., Yu C., Yin Z., Zhao X., Pan X., Yang H. (2021). Microstructure, optical, photoluminescence properties and the intrinsic mechanism of photoluminescence and photocatalysis for the BaTiO_3_, BaTiO_3_/TiO_2_ and BaTiO_3_/TiO_2_/CeO_2_ smart composites. Opt. Mater..

[36] Tvrdy K., Frantsuzov P.A., Kamat P.V. (2011). Photoinduced electron transfer from semiconductor quantum dots to metal oxide nanoparticles. Proc. Natl. Acad. Sci. USA.

[37] Mora-Sero I., Bisquert J., Dittrich T., Belaidi A., Susha A., Rogach A. (2007). Photosensitization of TiO_2_ layers with CdSe quantum dots: Correlation between light absorption and photoinjection. J. Phys. Chem. C.

[38] Wang X., Yu R., Wang P., Chen F., Yu H. (2015). Co-modification of F− and Fe (III) ions as a facile strategy towards effective separation of photogenerated electrons and holes. Appl. Surf. Sci..

[39] Zhang M., Shang Q., Wan Y., Cheng Q., Liao G., Pan Z. (2019). Self-template synthesis of double-shell TiO_2_@ ZIF-8 hollow nanospheres via sonocrystallization with enhanced photocatalytic activities in hydrogen generation. Appl. Catal. B Environ..

[40] Xu A.-W., Gao Y., Liu H.-Q. (2002). The preparation, characterization, and their photocatalytic activities of rare-earth-doped TiO_2_ nanoparticles. J. Catal..

[41] Nosaka Y., Nosaka A.Y. (2016). Kinetic processes in the presence of photogenerated charge carriers. Photocatalysis: Fundamentals and Perspectives.

[42] Xiang Q., Yu J., Wong P.K. (2011). Quantitative characterization of hydroxyl radicals produced by various photocatalysts. J. Colloid Interface Sci..

[43] Xiong S., Liu X., Zhu X., Liang G., Jiang Z., Cui B., Bai J. (2021). One-step preparation of well-dispersed spindle-like Fe_2_O_3_ nanoparticles on g-C_3_N_4_ as highly efficient photocatalysts. Ecotoxicol. Environ. Saf..

[44] Zhao W., Guo Y., Faiz Y., Yuan W.-T., Sun C., Wang S.-M., Deng Y.-H., Zhuang Y., Li Y., Wang X.-M. (2015). Facile in-suit synthesis of Ag/AgVO_3_ one-dimensional hybrid nanoribbons with enhanced performance of plasmonic visible-light photocatalysis. Appl. Catal. B Environ..

[45] Yu L., Huang Y., Yang Y., Xu Y., Wang G., Yang S. (2013). Photocatalytic degradation of organic dyes by H_4_SiW_6_Mo_6_O_40_/SiO_2_ sensitized by H_2_O_2_. Int. J. Photoenergy.

[46] Ning J., Wang M., Luo X., Hu Q., Hou R., Chen W., Chen D., Wang J., Liu J. (2018). SiO_2_ stabilized magnetic nanoparticles as a highly effective catalyst for the degradation of basic fuchsin in industrial dye wastewaters. Molecules.

