# Supplementary material for: Eu-Doped Zeolitic Imidazolate Framework-8 Modified Mixed-Crystal TiO2 for Efficient Removal of Basic Fuchsin from Effluent
Source: Materials (Basel). 2021 Nov 27;14(23):7265. doi: 10.3390/ma14237265 (PMC8658464; doi:10.3390/ma14237265)
Supplement: Supplementary file 1 [file materials-14-07265-s001.zip › materials-1426915-supplementary.pdf]

materials

MDPI

Supporting Information

Eu-Doped Zeolitic Imidazolate Framework-8 Modified Mixed-Crystal TiO_2 for Efficient Removal of Basic Fuchsin from Effluent

Wanqi Zhang [1,†], Hui Liu [1,†], Zhechen Liu [1], Yuhong An [1], Yuan Zhong [1], Zichu Hu [2], Shujing Li [1], Zhangjing Chen [3], Sunguo Wang [4], Xianliang Sheng [2], Xiaotao Zhang [2,5,*] and Ximing Wang [1,5,*]

[1] College of Material Science and Art Design, Inner Mongolia Agricultural University, Hohhot 010018, China; nmgndcyyzwq@emails.imau.edu.cn (W.Z.); nndcyylh@emails.imau.edu.cn (H.L.); liuzhechen@emails.imau.edu.cn (Z.L.); anyuhong@emails.imau.edu.cn (Y.A.); zhongyuan@email-simau.edu.cn (Y.Z.); lishujing@emails.imau.edu.cn (S.L.)
[2] College of Science, Inner Mongolia Agricultural University, Hohhot 010018, China; hzc101@emailLs.imau.edu.cn (Z.H.); shengxl@iccas.ac.cn (X.S.)
[3] Department of Sustainable Biomaterials, Virginia Polytechnic Institute and State University, Blacksburg, VA 24060, USA; chengo@vt.edu
[4] Sungro Bioresource & Bioenergy Technologies Corp, Alberta T6R3J6, Canada; wangsunguo@gmail.com
[5] Inner Mongolia Key Laboratory of Sandy Shrubs Fibrosis and Energy Development and Utilization, Hohhot 010018, China
* Correspondence: xiaotaozhang@imau.edu.cn (X.Z.); wangximing@imau.edu.cn (X.W.)
† Contributed equally to this paper.

1. Wavelength scanning curve of basic fuchsin

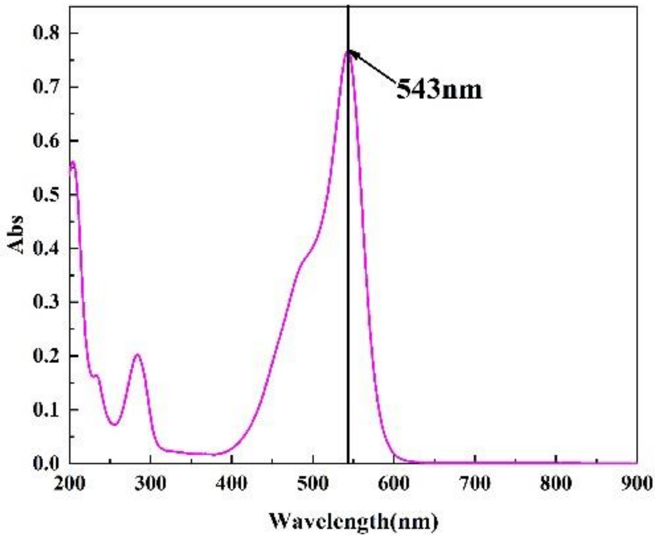

Figure S1. Wavelength scanning curve of basic fuchsin.

2. TiO_2 grain sizes at different temperatures

Table S1. TiO_2 grain sizes at different temperatures.

Sample	Half Band Width (β)	Bragg Diffraction Angle (2θ)	Bragg Semi-Diffraction Angle (θ)	Grain Size (D)
TiO_2 (350°C)	0.8096	25.27	12.635	16.99
TiO_2 (400°C)	0.7654	25.29	12.645	17.983
TiO_2 (500°C)	0.4954	25.25	12.625	27.748
TiO_2 (600°C)	0.2280	25.26	12.63	60.314

3. XRD patterns of ZIF-8 (Eu) @Mc-TiO_2doped with different contents of Eu

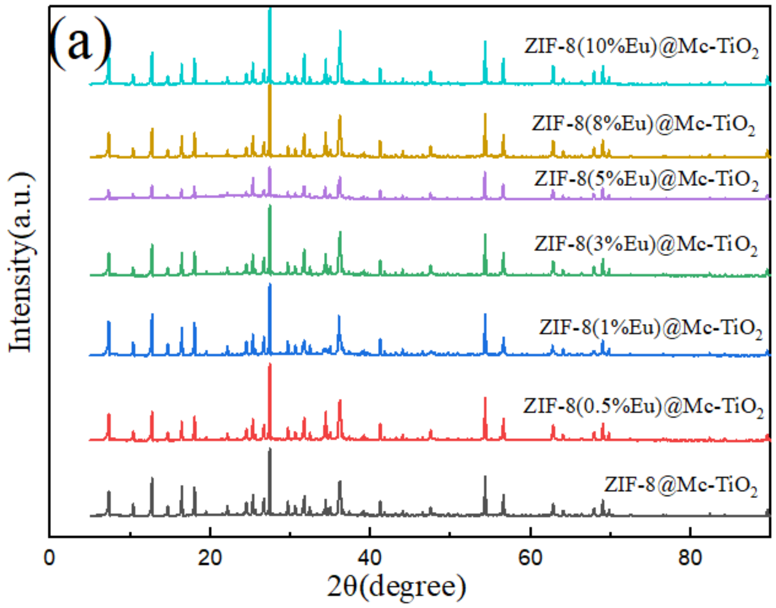

Figure S2. XRD patterns of ZIF-8 (Eu) @Mc-TiO_2 doped with different contents of Eu.
